# Root Ideotype Influences Nitrogen Transport and Assimilation in Maize

**DOI:** 10.3389/fpls.2018.00531

**Published:** 2018-04-24

**Authors:** Julie Dechorgnat, Karen L. Francis, Kanwarpal S. Dhugga, J. A. Rafalski, Stephen D. Tyerman, Brent N. Kaiser

**Affiliations:** ^1^Sydney Institute of Agriculture, School of Life and Environmental Sciences, The University of Sydney, Camden, NSW, Australia; ^2^School of Agriculture, Food and Wine, The University of Adelaide, Urrbrae, SA, Australia; ^3^Pioneer Hi-Bred International, Inc., Johnston, IA, United States; ^4^Genetic Discovery Group, DuPont Crop Genetics Research, DuPont Experimental Station, Wilmington, DE, United States

**Keywords:** *Zea mays*, nitrogen, gene expression, transport, root system architecture

## Abstract

Maize (*Zea mays*, L.) yield is strongly influenced by external nitrogen inputs and their availability in the soil solution. Overuse of nitrogen-fertilizers can have detrimental ecological consequences through increased nitrogen pollution of water and the release of the potent greenhouse gas, nitrous oxide. To improve yield and overall nitrogen use efficiency (NUE), a deeper understanding of nitrogen uptake and utilization is required. This study examines the performance of two contrasting maize inbred lines, B73 and F44. F44 was selected in Florida on predominantly sandy acidic soils subject to nitrate leaching while B73 was selected in Iowa on rich mollisol soils. Transcriptional, enzymatic and nitrogen transport analytical tools were used to identify differences in their N absorption and utilization capabilities. Our results show that B73 and F44 differ significantly in their genetic, enzymatic, and biochemical root nitrogen transport and assimilatory pathways. The phenotypes show a strong genetic relationship linked to nitrogen form, where B73 showed a greater capacity for ammonium transport and assimilation whereas F44 preferred nitrate. The contrasting phenotypes are typified by differences in root system architecture (RSA) developed in the presence of both nitrate and ammonium. F44 crown roots were longer, had a higher surface area and volume with a greater lateral root number and density than B73. In contrast, B73 roots (primary, seminal, and crown) were more abundant but lacked the defining features of the F44 crown roots. An F1 hybrid between B73 and F44 mirrored the B73 nitrogen specificity and root architecture phenotypes, indicating complete dominance of the B73 inbred. This study highlights the important link between RSA and nitrogen management and why both variables need to be tested together when defining NUE improvements in any selection program.

## Introduction

Nitrogen is an essential macronutrient required for plant growth. It is a primary constituent of nucleic acids, amino acids and proteins. nitrogen is also an important signaling molecule influencing a large number of plant processes including lateral root growth, resistance to biotic and abiotic stress, regulation of seed germination and mediation of hormone signaling (Vidal et al., 2010; Alvarez et al., 2012; Wang et al., 2012; Xu et al., 2012).

Under most cropping situations, plant productivity is limited by the availability of nitrogen in the soil. Nitrogen deficiency generally leads to a reduction in total biomass and harvestable yield, which directly impacts grower’s profitability (Makino, 2011; Sinclair and Rufty, 2012). To compensate, growers apply inorganic nitrogen fertilizers at rates often dictated by expected yields and best practice for the region and the crop. Mismanagement or poorly timed applications of nitrogen-fertilizers can result in unintended nitrogen pollution (Sebilo et al., 2013). Soil microbes can produce nitrogen oxides, potent greenhouse gasses that can escape into the atmosphere. Moreover, excessive rainfall or irrigation events cause significant nitrate leaching from the soil profile (Lassaletta et al., 2014) regardless of nitrogen fertilizer use, cropping system, or native ground cover. This causes contamination of ground water and excessive algal growth in rivers and deltas leading to eutrophication and subsequently death of aquatic life.

Higher plants use organic nitrogen, ammonium and nitrate as nitrogen sources. However, access to organic nitrogen is often impeded by a competition with soil microorganisms. Nitrate and ammonium are then the main forms of nitrogen available to roots although ammonium content can be limited by the soil type and microorganism flora (Bloom, 2015). The uptake of nitrate and ammonium into roots involves two physiological mechanisms (Glass et al., 2002). At elevated concentrations (>250 μM), a low-affinity high capacity transport system (LATS) is active. When nitrogen concentrations are lower (<250 μM), a high-affinity low-capacity transport system (HATS) dominates. The uptake of nitrate is under the genetic control of the *NPF* (*NRT1/PTR*) and *NRT2* families of nitrate transporter genes encoding the LATS and HATS proteins, respectively (Dechorgnat et al., 2011). The nitrate HATS requires the presence of companion proteins, NRT3 (NAR2), for the NRT2 proteins to be active (Okamoto et al., 2006; Dechorgnat et al., 2011; Pii et al., 2016). The uptake of ammonium at low external concentrations (HATS) involves members of the *AMT* (AMmonium Transporter) gene family (Yuan et al., 2007). At present there is no known transporter for the ammonium LATS activity in plants, although the recent identification of AMF1 proteins in soybean and yeast highlight a promising candidate for this transport phenomenon (Chiasson et al., 2014). Once inside root cells, both nitrate and ammonium ions can be assimilated directly or stored intracellularly within the vacuole and/or cytoplasm of root cells (Dechorgnat et al., 2011; Xu et al., 2012). Nitrate and ammonium can also undergo radial transport to the xylem using a combination of symplastic and apoplastic pathways.

The assimilation of nitrate (root or shoot-based processes) involves a reduction to nitrite by the cytosolic enzyme, nitrate reductase (NR) (Fischer et al., 2005). In both root and shoot tissues nitrite is further reduced to ammonium by a plastid localized nitrite reductase (NiR) (Miflin, 1974; Wray, 1993). The ammonium generated from nitrate reduction, together with ammonium accumulated by direct root uptake, is mainly assimilated in the plastid by the glutamine synthetase-glutamate synthase (GS-GOGAT) cycle where GS facilitates the amination of glutamate to form glutamine (Cren and Hirel, 1999; Miflin and Habash, 2002). Glutamine reacts subsequently with the 2-oxoglutarate to form two molecules of glutamate through the activity of GOGAT (Suzuki and Knaff, 2005). Following the generation of both glutamine and glutamate via the GS-GOGAT cycle, assimilated nitrogen can then enter a range of amination or deamination reactions leading to the production of amino acids. Alanine aminotransferase, a reversible enzyme, has been reported to play a key role in regulating the glutamate levels in maize leaves (Trucillo Silva et al., 2017).

In this work, we characterize two contrasting maize inbred lines, F44 and B73 each independently selected on diverse soil and growing conditions from two locations in the United States. B73 (release 1973) is a common inbred in the mid-maturity zone selected on rich mollisol soils in Iowa. F44 is an older inbred (release 1944) from the late-maturity group selected in Florida on predominantly sandy acidic soils. Our results indicate root presentation and function differentiates B73 from F44. The lines display contrasting nitrogen acquisition strategies influenced by root architecture, nitrogen transport activities (nitrogen specificity) and assimilation patterns. The development of a hybrid between F44 and B73 revealed a genetic predisposition toward increased root branching and ammonium acquisition strategies, potentially at the expense of enhanced nitrate transport and assimilation capacity.

## Results

### Root Nitrogen Uptake, Storage and Assimilation

**FIGURE 1:**
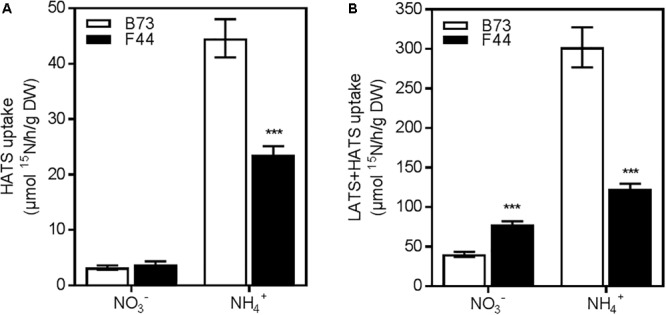
Nitrogen uptake capacity of B73 (white) and F44 (black) root systems. **(A)** High affinity transport (HATS) of 50 μM of nitrate (NO_3_^-^) or ammonium (NH_4_^+^). **(B)** Low affinity transport (LATS+HATS) of 2.5 mM of nitrate (NO_3_^-^) or ammonium (NH_4_^+^). Values are means (±SE) from six individual plants. Similar results were obtained in three other independent experiments. Asterisks indicate significant differences between lines at ^∗∗∗^*P* < 0.005 (Student’s *t*-test).

**FIGURE 2:**
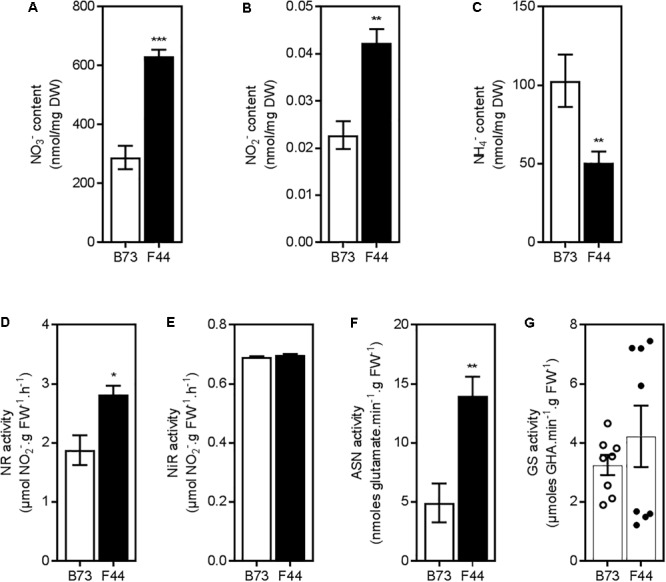
Inorganic nitrogen content and enzymatic activities in roots of B73 (white) and F44 (black) maize inbreds. Measurements of nitrate **(A)**, nitrite **(B)**, and ammonium content **(C)**. Values are means (±SE) from 4 to 8 individual plants. Similar results were obtained in two other independent experiments. Analysis of nitrate reductase activity **(D)**, nitrite reductase activity **(E),** and asparagine synthetase activity **(F)**. Values are means (±SE) from four individual plants. Similar results were obtained in another independent experiment. **(G)** Measurements of glutamine synthetase activity in B73 (open circles) and F44 (closed circles) in eight individual plants from two independent experiments, histograms represent the mean values (±SE). Asterisks indicate significant differences between lines at ^∗^*P* < 0.05, ^∗∗^*P* < 0.01, ^∗∗∗^*P* < 0.005 (Student’s *t*-test).

To determine the nitrate or ammonium transport activities of B73 and F44 root systems, we grew plants for 3 weeks in an ebb and flow hydroponic system at adequate nitrogen levels (2.5 mM NH_4_NO_3_), as previously described by Garnett et al. (2013), and then measured their constitutive N uptake capacities across both the HATS (50 μM N) and LATS+HATS (2.5 mM N) systems. We used NH_4_NO_3_ as a nitrogen source to balance the available nitrate and ammonium ions presented to the root system at a concentration that supports good growth of maize in hydroponic systems. The constitutive nitrate HATS was similar between the two inbreds, whereas B73 displayed (*P* < 0.005) 1.9-fold higher ammonium HATS activity than F44 (**Figure 1A**). Elevated ammonium influx continued into the LATS+HATS range (**Figure 1B**, *P* < 0.005), where it was 2.4-fold higher in B73 than F44. In contrast, the LATS+HATS for nitrate uptake was 1.9-fold lower in B73 than in F44 (**Figure 1B**, *P* < 0.005). Overall, the two inbreds displayed a higher capacity for ammonium transport (unidirectional influx) than that of nitrate across both the HATS and the LATS activity ranges. The higher rate of LATS+HATS nitrate flux in F44 was reflected in significantly greater accumulated root nitrate (*P* < 0.005) and nitrite (*P* < 0.01) content (**Figures 2A,B**). Like the observed flux rates, B73 roots had more ammonium (*P* < 0.01) than those of F44 (**Figure 2C**) by a difference of twofold.

*In vitro* NR activity (NRA) was measured to identify if differences in nitrate assimilation pathways exist in F44 and B73 roots. In F44 roots, NRA was ∼1.5-fold higher than B73 (**Figure 2D**, *P* < 0.05). The higher NR activity in F44 is consistent with its elevated total root nitrate (2.2-fold) and nitrite (1.9-fold) contents (**Figures 2A,B**, *P* < 0.01). NiR activity was similar between the two inbreds (**Figure 2E**). Asparagine synthetase (ASN) activity was higher in F44 (2.8-fold) than B73 (**Figure 2F**, *P* < 0.01). GS activity was similar between the inbreds although it was highly variable in F44 (**Figure 2G**).

### Transcriptional Analysis of N Transport and Assimilatory Pathways

**FIGURE 3:**
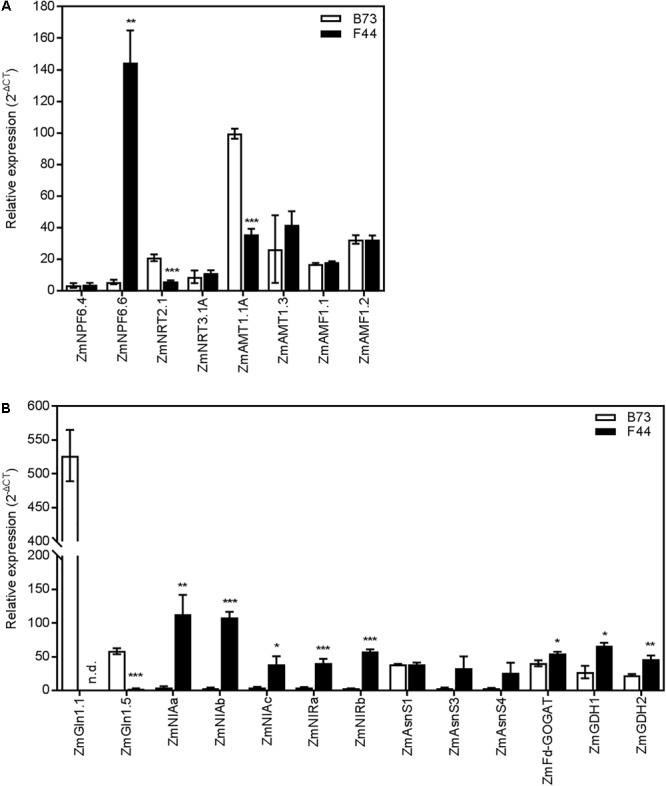
Gene expression in B73 (white) and F44 (black) roots. **(A)** Expression of nitrogen transporter genes. **(B)** Expression of nitrogen metabolism pathway genes. Values are means (±SE) from three individual plants. Similar results were obtained in another independent experiment. Asterisks indicate significant differences between lines at ^∗^*P* < 0.05, ^∗∗^*P* < 0.01, ^∗∗∗^*P* < 0.005 (Student’s *t*-test). NPF/NRT, nitrate transporter; AMT, ammonium transporter; AMF, ammonium facilitator; GLN, glutamine synthetase; NIA, nitrate reductase; NIR, nitrite reductase; AsnS, asparagine synthetase; GOGAT, glutamate synthase; GDH, glutamate dehydrogenase; n.d., non-detectable.

We investigated the expression patterns of genes encoding N transport and assimilation pathways in both inbreds (**Supplementary Table S1**). In *Arabidopsis thaliana*, AtNPF6.3 (AtNRT1.1) has been shown to be one of the main contributors to both high and low-affinity nitrate uptake (Huang et al., 1996; Liu et al., 1999). In maize, *AtNPF6.3* has four putative homologs (Plett et al., 2010): *ZmNPF6.4* (*ZmNRT1.1A*), *ZmNPF6.6* (*ZmNRT1.1B*), *ZmNPF6.7* (*ZmNRT1.1C*), and *ZmNPF6.5* (*ZmNRT1.1D*). The expression of *ZmNPF6.4* was similar between B73 and F44 (**Figure 3A**). However, there was a clear difference in the expression pattern of *ZmNPF6.6*, which was significantly higher (*P* < 0.01) in F44 than B73 (**Figure 3A**). The remaining two homologs, *ZmNPF6.7* and *ZmNPF6.5*, were not expressed in roots under the conditions used in our experiments (data not shown). A new family of putative low affinity ammonium transporters, *AMF1*, has been recently discovered in soybean (Chiasson et al., 2014). There was no change in expression of the two *AMF1* homologs, *ZmAMF1.1* and *ZmAMF1.2* between the two inbred lines (**Figure 3A**).

We also investigated the expression patterns of selected genes involved in nitrate and ammonium HATS activity. *ZmNRT2.1*, a maize homolog of the Arabidopsis high-affinity nitrate transporter (*AtNRT2.1*) (Filleur et al., 2001; Plett et al., 2010) was more abundant (*P* < 0.005) in B73 than F44 (**Figure 3A**). In contrast, *ZmNRT3.1A*, a maize homolog of *AtNRT3.1* (Plett et al., 2010), was found expressed similarly in both inbreds (**Figure 3A**). No expression of its homolog *ZmNRT3.1B* could be detected in our experiments (data not shown). Gu et al. (2013) recently showed that the main contributors to maize root high-affinity ammonium transport were most likely *ZmAMT1.1A* and *ZmAMT1.3*. We found that the expression of *ZmAMT1.1A* was increased by 2.8-fold in B73 compared to F44 (*P* < 0.005) and *ZmAMT1.3* roughly equal between the two inbreds (**Figure 3A**).

To look for differences in N assimilatory pathways between inbreds, representative genes linked to nitrate and ammonium assimilation were selected for qPCR analysis. NR is represented by four putative genes in maize, *ZmNIAa, ZmNIAb, ZmNIAc*, and *ZmNIAd* (Gramene^1^). *ZmNIAa, ZmNIAb*, and *ZmNIAc* were all expressed at a significantly (*P* < 0.05) higher level in F44 than in B73 (**Figure 3B**). The transcript for *ZmNIAd* was undetectable in both the inbreds (data not shown). Two genes potentially coding for NIR, *ZmNIRa* and *ZmNIRb*, were also characterized (Gramene^1^). Similar to NR, both NIR genes were expressed at a higher level (10- and 20-fold, respectively) in F44 than in B73 (**Figure 3B**).

Downstream assimilation of ammonium by GS was also investigated. GS is encoded by six genes in maize: the cytosolic GS, *ZmGLN1.1* to *ZmGLN1.5* and the single plastidic GS, *ZmGLN2* (Martin et al., 2006). *ZmGLN1.1* and *ZmGLN1.5* were strongly expressed in B73 roots (**Figure 3B**). *ZmGLN1.1* transcript, which was highly abundant in B73, was undetectable in F44. Similarly, the expression of *ZmGLN1.5* was significantly reduced (*P* < 0.005) in F44 relative to B73 (**Figure 3B**). We found no differences in expression between the inbreds for the other four GS homologs (*ZmGLN1.2, ZmGLN1.3, ZmGLN1.4*, and *ZmGLN2*) (**Supplementary Figure S1**). The single gene encoding ferredoxin dependent glutamate synthase (*ZmFd-GOGAT*) was also studied (Sakakibara et al., 1992). Its expression in F44 was 1.4-fold higher than in B73 (**Figure 3B**). ASN is encoded by four genes in maize, *ZmAsnS1, ZmAsnS2, ZmAsnS3*, and *ZmAsnS4* (Todd et al., 2008). Although not significant, there was a trend toward higher levels of expression of *ZmAsnS3* and *ZmAsnS4* in F44 than in B73 (**Figure 3B**). *ZmAsnS1* was expressed at the same level between the two inbreds. We were unable to amplify *ZmAsnS2* in either inbred (data not shown). Expression of the two genes encoding glutamate dehydrogenase, *ZmGDH1* and *ZmGDH2* (Sakakibara et al., 1995), were twofold higher in F44 than in B73 (**Figure 3B**).

### Contrasting Root Phenotypes of B73 and F44

**FIGURE 4:**
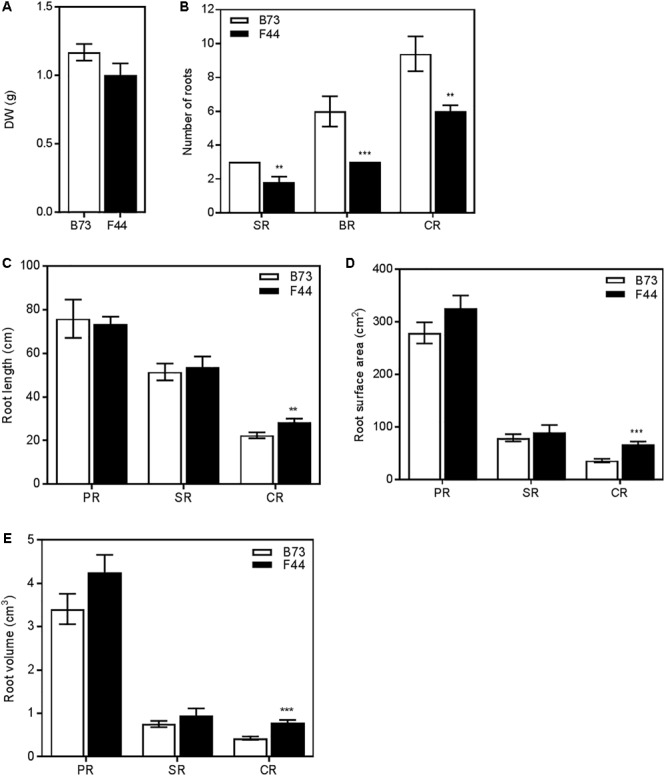
Root architecture measurements of B73 (white) and F44 (black) plants grown on sand. **(A)** Total root system dry weights. **(B)** Number of seminal roots (SR), brace roots (BR) and crown roots (CR). **(C)** Individual root lengths. **(D)** Individual root surface areas. **(E)** Individual root volumes. Values are means (±SE) from five individual plants. Similar results were obtained in another independent experiment. Asterisks indicate significant differences between lines at ^∗∗^*P* < 0.01, ^∗∗∗^*P* < 0.005 (Student’s *t*-test).

We grew the two inbred lines for 4 weeks in 1 m high PVC pipes (100 mm diameter) filled with a mixture of sand and diatomite and their root morphologies recorded. Total root dry weight was similar between the two lines (**Figure 4A**). Further examination of the number of root types showed that F44 had fewer seminal roots, brace roots and crown roots than B73 (**Figure 4B**, *P* < 0.01). As the role of brace roots is mainly structural in younger 4-week-old plants (Hochholdinger et al., 2004), their morphology was not recorded in this analysis. The primary and seminal root lengths, surface areas and volumes were similar between lines (**Figures 4C–E**). However, crown root lengths, surface areas and volumes were higher in F44. F44 crown roots were ∼1.3-fold longer than B73 (**Figure 4C**, *P* < 0.01) and the surface area and volume ∼1.8-fold that of B73 (**Figures 4D,E**, *P* < 0.005).

**FIGURE 5:**
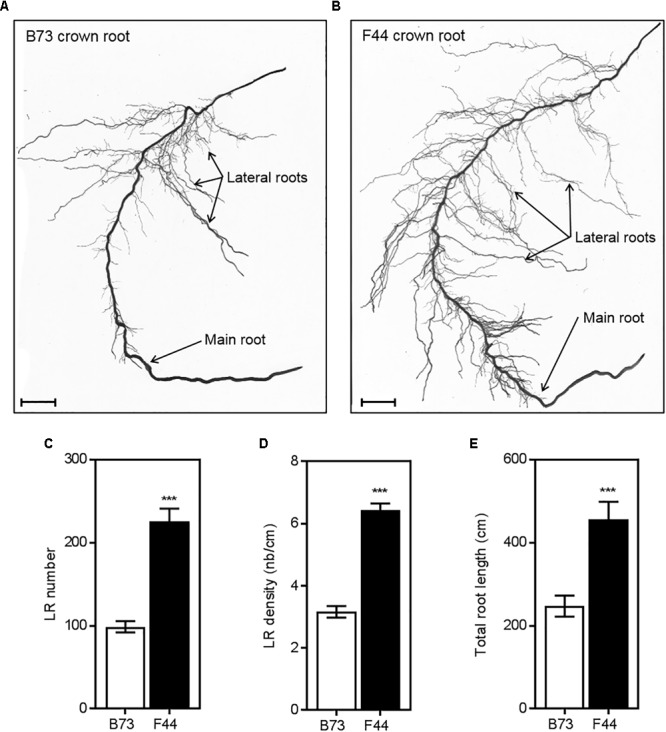
Comparison of crown root architecture of B73 **(A)** and F44 **(B)** maize inbreds. Measurements of lateral root numbers **(C)**, lateral root densities **(D)** and total root lengths **(E)** of B73 (white) and F44 (black) crown roots. Values are means (±SE) from five individual plants. Similar results were obtained in another independent experiment. Scale bars = 25 mm. Asterisks indicate significant differences between lines at ^∗∗∗^*P* < 0.005 (Student’s *t*-test).

Representative scanned images of the B73 and F44 crown root phenotypes are shown in **Figures 5A,B**, respectively. The average number of lateral roots per crown root (**Figure 5C**) was lower in B73 (twofold reduced lateral root density, 3.2/cm) than F44 (6.4 lateral root/cm per crown root) (**Figure 5D**). Average crown root total root length (main root plus laterals) was 1.84 times longer than B73 (**Figure 5E**).

### Root Specific Nitrate and Ammonium Uptake Activities

**FIGURE 6:**
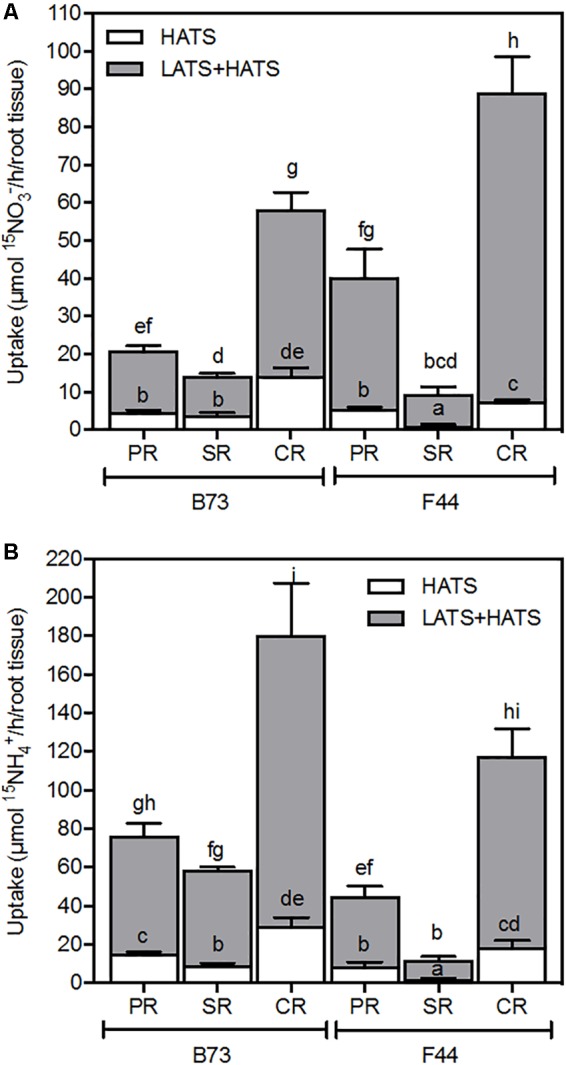
Nitrate **(A)** and ammonium **(B)** uptake capacity of B73 and F44 in the PR, SR, and CR root tissues. Values are means (±SE) from five individual plants. Different letters indicate significant differences among means at *P* < 0.05 (ANOVA).

We examined the transport activities and associated gene expression properties of individual root components. As previously seen in **Figure 1**, the rate of nitrate uptake was higher in F44 inbred than B73, while B73 continued to show a preference for ammonium (**Figures 6A,B**, respectively). Although all root types accumulated nitrate and ammonium, it appears the crown roots contributed to the majority of net nitrogen uptake across both the HATS and LATS+HATS ranges for both inbreds (**Figures 6A,B**). Interestingly, the seminal roots were the least active for nitrogen uptake in both B73 and F44 (**Figures 6A,B**).

**FIGURE 7:**
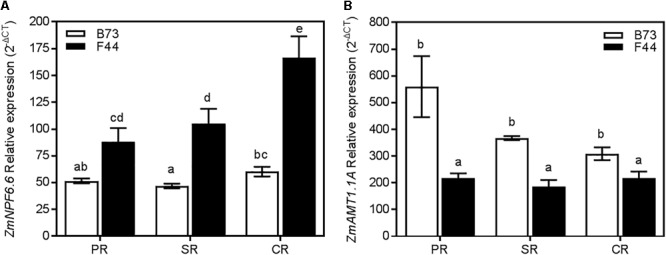
Expression of *ZmNPF6.6* **(A)** and *ZmAMT1.1A* **(B)** in B73 (white), and F44 (black) in the PR, seminal SR, and CR root tissues. Values are means (±SE) from 4 to 5 individual plants. Different letters indicate significant differences among means at *P* < 0.05 (ANOVA).

We earlier showed that *ZmNPF6.6* and *ZmAMT1.1A* were the main differently expressed nitrogen transport genes in F44 and B73 roots (**Figure 3A**). Across all three root types (primary, seminal, and crown) *ZmNPF6.6* expression was higher in F44 than B73 (**Figure 7A**). In contrast, *ZmAMT1.1A* transcripts were more significant in B73 roots (**Figure 7B**). *ZmNPF6.6* expression didn’t vary across root types of B73, while in F44, expression increased by 1.89- and 1.58-fold in the crown roots compared to the primary and seminal roots, respectively (**Figure 7A**). Although not significant, *ZmAMT1.1A* expression was higher in B73 primary roots than in the seminal and crown roots (**Figure 7B**). In F44, the expression of *ZmAMT1.1A* was similar between root types (**Figure 7B**). The expression of *ZmNRT2.1* and its partner *ZmNRT3.1A* were also measured in the different root types. Although the genes presented differences of expression between genotypes, no root specific expression was detected (**Supplementary Figure S2**).

### B73 × F44 Hybrid Performance

**FIGURE 8:**
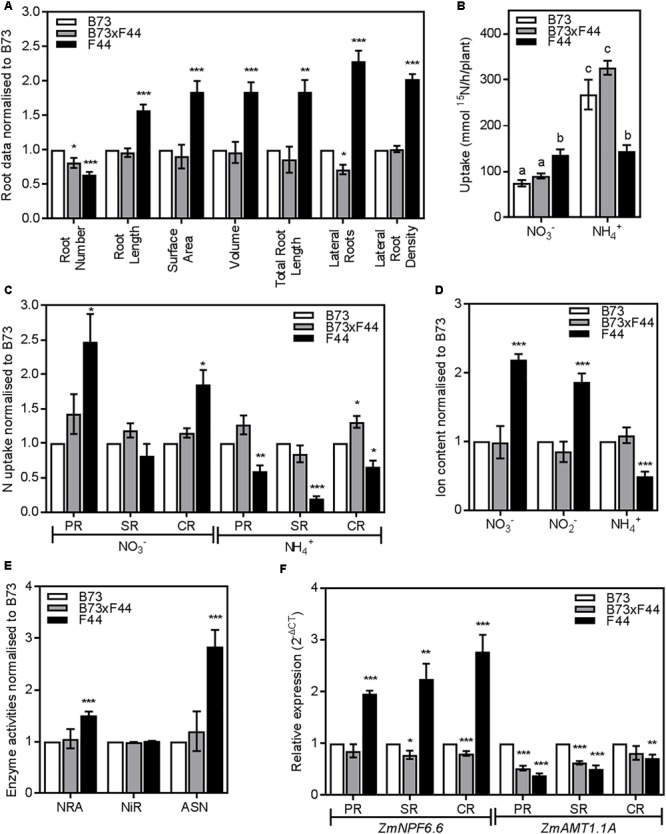
Phenotypic comparison of F1 BK3 (gray) and parental B73 (white) and F44 (black) roots. **(A)** Crown root measurements normalized to B73. **(B)** Low affinity transport (LATS+HATS) of 2.5 mM of nitrate (NO_3_^-^) and ammonium (NH_4_^+^). **(C)** NO_3_^-^ and NH_4_^+^ LATS+HATS capacities in the PR, SR, and CR root tissues normalized to B73. **(D)** Measurements of nitrate (NO_3_^-^), nitrite (NO_2_^-^), and ammonium content (NH_4_^+^) normalized to B73. **(E)** Analysis of nitrate reductase activity (NRA), nitrite reductase activity (NiR), and asparagine synthetase activity (ASN) normalized to B73. **(F)** *ZmNPF6.6* and *ZmAMT1.1A* gene expression in the PR, SR, and CR root tissues normalized to B73. Values are means (±SE) from 3 to 8 individual plants. Asterisks indicate significant differences with B73 at ^∗^*P* < 0.05, ^∗∗^*P* < 0.01, ^∗∗∗^*P* < 0.005 (Student’s *t*-test). Different letters indicate significant differences among means at *P* < 0.05 (ANOVA).

A F1 hybrid (B73 × F44) was generated from a cross between B73 and F44. When grown alongside the parental lines, F44 and B73, B73 × F44 presented a similar root structure to B73. The analysis of B73 × F44 seminal and primary roots revealed a similar architecture pattern to both B73 and F44, although F44 developed fewer seminal roots compared to the two other lines (**Supplementary Figures S3A,B**). The detailed analysis of the CR showed that B73 × F44 root morphology was much the same as B73 (**Figure 8A**). Indeed, where F44 crown roots were longer with a larger surface area, volume, total root length and LR density, B73 × F44 behaved like B73. B73 × F44 presented a lower number of crown roots than B73 but higher than F44. The other notable difference between B73 × F44 and its parents was in the LR number which was reduced in the hybrid compared to B73 and F44 (**Figure 8A**).

We grew B73 × F44 under the same conditions as the parents and examined NO_3_^-^ and NH_4_^+^ transport properties. B73 × F44 plants displayed NO_3_^-^ and NH_4_^+^ uptake properties similar to the B73 parent both in the HATS (data not shown) and the LATS+HATS (**Figure 8B**) concentration ranges. B73 × F44 NO_3_^-^ uptake across the different root types were similar to B73 (**Figure 8C**). PR and SR of B73 × F44 displayed comparable ammonium uptake with B73, while B73 × F44 crown roots took up more ammonium than the crown roots of both parents (**Figure 8C**).

Correspondingly, nitrate, nitrite and ammonium contents in the roots of the hybrid mirrored the B73 parental line (**Figure 8D**). There was significantly less nitrate and nitrite and more ammonium (*P* < 0.005) stored in the roots of B73 and B73 × F44 relative to F44. The hybrid displayed NRA and ASN activities similar to the B73 parental line (**Figure 8E**).

In B73 × F44, none of the studied genes followed the pattern of F44 expression. Indeed, most of the B73 × F44 transcripts resembled expression profiles of B73 (**Supplementary Figure S4**). The exception was *ZmFd-GOGAT* which was significantly less expressed in B73 × F44 than in B73. Examination of *ZmNPF6.6* and *ZmAMT1.1A* expression in the different root types revealed differences between the lines (**Figure 8F**). *ZmNPF6.6* showed reduced expression in B73 × F44 seminal and crown roots (*P* < 0.05) compared to both parents, although primary root gene expression was similar between B73 × F44 and B73. The expression of *ZmAMT1.1A* in the crown roots was similar in B73 and B73 × F44, while it was significantly decreased in F44 (*P* < 0.01). Both B73 × F44 and F44 showed reduced levels of *ZmAMT1.1A* transcripts (*P* < 0.005) in their primary and seminal roots compared to B73 (**Figure 8F**).

## Discussion

### Contrasting Nitrate and Ammonium Transport Preferences Between Lines

Our results show that B73 and F44 have contrasting root N transport pathways during their vegetative stages of growth. The phenotypes were dependent on the form and concentration of N supplied and in their manner of root presentation. B73 clearly showed a greater capacity for ammonium transport than F44 (*P* < 0.005); in contrast, F44 displayed an enhanced capacity for nitrate uptake (*P* < 0.005). Transport kinetics were supported by differential expression of select genes encoding nitrate (*ZmNPF6.6*) and ammonium (*ZmAMT1.1*) transporters in F44 and B73, respectively.

Nitrate transport in plants has been linked with the activity of the *NPF6/NRT1.1/CHL1* family of transport proteins (Dechorgnat et al., 2011). In maize, four homologs of *AtNPF6.3/NRT1.1/CHL1* have been categorized (Plett et al., 2010). Of these only two (*ZmNPF6.4* and *ZmNPF6.6*) were found expressed in roots. Recently, we have functionally characterized ZmNPF6.4 and ZmNPF6.6, confirming both transport nitrate at high external nitrate concentrations (LATS), while ZmNPF6.6, a nitrate inducible protein, also shows activity at low concentrations (HATS) with monophasic nitrate uptake kinetics (Km ∼ 200 μM) across both the HATS and LATS concentration range (Wen et al., 2017). ZmNPF6.6 was significantly upregulated in F44 roots (*P* < 0.01) which was correlated with an increased pool of stored nitrate in these tissues. In contrast, the repressed expression of *ZmNPF6.4* and *ZmNPF6.6* in B73 was mirrored by a reduction in nitrate LATS uptake capacity and reduced tissue nitrate content. In the high-affinity range, *ZmNRT2.1*, a maize homolog of *AtNRT2.1* (Filleur et al., 2001; Plett et al., 2010) was found to be significantly more abundant in B73 than in F44 (*P* < 0.005). It has been recently shown that ZmNRT2.1 and the accessory protein ZmNRT3.1A form a 150 kDa oligomer which is the main component of the HATS (Pii et al., 2016). The analogous expression pattern of *ZmNRT3.1A* between B73 and F44 may explain the similar high-affinity nitrate uptake displayed by the inbreds. Another possibility is that the enhanced expression patterns of *ZmNPF6.6* in F44 and *ZmNRT2.1* in B73 may normalize HATS activities between the two lines. Their relative contribution of each protein to HATS nitrate uptake still needs to be defined. In F44, *ZmNPF6.6* root transcription was higher in crown than in seminal and primary roots. We assume this expression pattern may be linked to the elevated nitrate uptake ability of the crown root tissues. However, the expression of *ZmNPF6.6* in all B73 root tissues was comparable even though the crown roots were found to be more active in nitrate uptake. Further investigation into the individual roles of ZmNRT2.1 and ZmNPF6.6 in nitrate HATS activity is required.

B73 consistently displayed higher ammonium uptake capacity than F44, an activity which we believe translated into higher levels of stored ammonium in the B73 root tissues. B73 roots also had elevated expression of GS enzyme genes (*ZmGln1.1* and *ZmGln1.5*). Previous studies have indicated *ZmAMT1.1A* is a primary high-affinity ammonium transporter expressed in maize roots (Gu et al., 2013). Our data would support this observation as *ZmAMT1.1A* expression was 2.8-fold higher in B73 than F44. This increased activity may explain in part the higher levels of ammonium HATS in B73 than F44 and the differences in *ZmGlns* expression. The molecular identity of the ammonium LATS has yet to be characterized in plants. Candidate genes have recently been discovered including members of the *AMF1* gene family common to both plants and yeast (Chiasson et al., 2014). We examined the expression of two identified AMF maize homologs, *ZmAMF1.1* and *ZmAMF1.2*. Both genes were expressed at a similar level in the roots of both inbreds (*P* > 0.05) suggesting that ZmAMT1.1A is likely a key contributor to the enhanced ammonium transport and accumulation in B73 roots. However, the role of each root type requires further investigation as we found crown roots to have high ammonium uptake capacity in B73 but lower *ZmAMT1.1A* expression than that of the primary roots.

### Root Ideotypes for Improved NUE?

The quest for improved nitrogen use efficiency (NUE) traits has led to numerous studies on the physiology and genetics of how maize roots access, transport and then utilize accumulated nitrogen (Martin et al., 2006; Good et al., 2007; Garnett et al., 2009, 2013; Cañas et al., 2010; McAllister et al., 2012; Haegele et al., 2013; Simons et al., 2014; Zamboni et al., 2014; Trucillo Silva et al., 2017). Underlying this research is the inherent plasticity of the root system to respond to nutritional change to better access the soil profile for mining nitrogen. This is particularly important across the growth cycle as soil nitrogen availability ultimately changes during the season from localized depletion, runoff, and leaching. The underlying genetic control of maize roots to respond to altered soil N profile is poorly understood (Kong et al., 2014; Rogers and Benfey, 2015).

It is well acknowledged that nutrient availability in the soil can influence both root growth and the pattern of root development. As N becomes available there is a general trend for shoot to root ratios to decrease (Marschner, 2011). Localized nutrient provision can also influence spatial changes to root development, in the form of increased root branching as nutrient patches are encountered (Drew, 1975; Zhang and Forde, 1998; López-Bucio et al., 2003). The process of how N activates root primordial growth, and the internal mechanisms used to regulate growth thereafter is thought to involve multiple levels of genetic and metabolic responsive strategies linked to nitrate perception, transport and assimilation as summarized in recent reviews by Giehl and von Wirén (2014), Krapp et al. (2014), Medici and Krouk (2014). One would predict that over a growing season the sum response to spatial differences in nutrient availability in the soil relative to plant demand defines the overall root ideotype. Whether this is the case is debatable as Gaudin et al. (2011) showed that aeroponically grown maize roots respond to reduced N supply across the root system by changing their root system architecture (RSA), favoring longer but fewer crown roots and a significant increase in the lateral to crown root ratio. In this study, we observed contrasting responses in RSA between B73 and F44 when nutrient supply (2.5 mM NH_4_NO_3_) was homogeneous across the root system using a hydroponic system or with traditional sand grown pot plants. In general, B73 presented a higher density root system (brace, seminal, and crown roots per plant) than F44, which predominantly altered and increased its crown root profile (length, surface area and volume). According to Hochholdinger et al. (2004), shoot-borne crown roots dominate the maize root system and are responsible for the majority of water uptake, possibly through higher xylem suction than that of seminal roots (Doussan et al., 1998). Our study revealed that all roots types were involved in nitrogen uptake but the crown root system was the main nitrogen supplier of the root system. Our findings are in agreement with previous studies describing the link between crown root and nitrogen uptake. Saengwilai et al. (2014) showed that reduced numbers of crown roots in maize was correlated with longer individual crown roots and higher nitrate uptake and higher nitrogen content. Whereas, increased crown root numbers resulted in shorter (shallow) root systems with less effective nitrate uptake capacity. Our study supports this trend, as F44 displayed a lower number of crown roots per plant but their increased length and lateral root density allowed for higher nitrate uptake than B73, which had more but shorter crown roots with a lower lateral root density. As nutrient provision was homogeneous across the root system, our data suggests a potential correlation between plant nitrogen preference and RSA, which most likely would underlie any influence of secondary spatial interactions between the root and the soil profile.

The B73 root ideotype consisted of more roots (seminal, brace, and crown) per plant, where crown roots were shorter and had lower lateral root density than F44. Like nitrate, the highest rate of ammonium transport occurred in the crown roots followed by primary and seminal roots, respectively. It would appear B73’s advantage in ammonium transport over F44 was through the role of the primary and seminal roots where uptake rates were higher at concentrations extending across both the HATS and LATS activity ranges. In Arabidopsis roots, the high-affinity ammonium transporters, AtAMT1s, are predominantly found in root hairs, root epidermal cells, cortical cells, and endodermal cells in primary and lateral roots (Yuan et al., 2007). According to the observed phenotype in B73, one would presume a similar location of ZmAMT1s in maize although this hypothesis needs to be verified.

The contrasting RSA between B73 and F44 could be related to their area of origin. F44 is a yellow dent maize inbred originally selected at the Florida Agricultural Experiment Station (Flint-Garcia et al., 2005). Soil types in this region are variable but predominantly consist of spodosols and ultisols (Effland et al., 2006). These soil types are mainly acidic, with poor nutrient status as a result of excessive leaching. Soils such as these often favor a reduction in crown root density in exchange for an increase in the length and the density of the first-order and second order LRs that help reduce root competition and increase soil exploration capacity (Kong et al., 2014). In contrast, B73 is an inbred selected in Iowa, which is rich in mollisol soils (Effland et al., 2006). Mollisols are traditionally deep surface soils with high organic matter and nutrient content. Typically, fertile soils reduce the need for soil foraging of roots to access nutrients and water. In general, crops grown under these conditions (i.e., B73 as observed in our experiments) produce short but dense and thick root systems (Kong et al., 2014; Postma et al., 2014; York et al., 2015; Zhan and Lynch, 2015).

Although our experiments were not conducted under field conditions, the inbreds exhibited divergent root architectures, which suit the conditions where they originated from. Interestingly, although root shape varied, the two root systems shared similar dry weights suggesting carbon allocation to the overall root profile was not a discerning feature between the two inbreds.

### Differences in Root Nitrogen Assimilation

In this study, we observed that F44 roots showed higher levels of total NR and asparagine synthetase (ASN) activities relative to B73 (*P* < 0.05). Enhanced root NR and ASN activities have been observed in selected maize lines, including those bred for high seed protein content (Illinois High Protein – IHP) (Lohaus et al., 1998). We assume the elevated NR and ASN activities in F44 roots is in response to their enhanced nitrate transport activities and associated gene expression patterns that highlight nitrate transport and assimilation. Whether this is a metabolic niche of F44 remains to be seen but it does suggest an organization of both transport and assimilatory activities that promote a greater involvement of the root system to overall plant nitrate acquisition and metabolism. This assumption is supported by observations of DeBruin et al. (2013), which highlighted the important contribution that root activity (uptake and metabolism) has on maize N distribution, particularly at later stages (post flowering) to meet nitrogen demand in developing maize kernels. The contrasting root morphologies displayed between F44 and B73 may be an important phenotype that supports this requirement. Future work is now required to examine shoot-based N assimilatory pathways between these two lines and how this relates to the root phenotypes observed in this study.

The expression profiles of other N assimilatory genes were also investigated. Whilst all the GS genes were expressed in the roots, only *ZmGln1.1* and *ZmGln1.5* differed between the inbreds. No transcript of *ZmGln1.1* was detected in F44 whereas its expression in B73 was the highest across all the whole gene family. *ZmGln1.1* encodes for the cytosolic GS1.1 and is specific to the roots in maize (Li et al., 1993; Martin et al., 2006). *ZmGln1.5*, another gene that differed in expression between the two inbred lines, also codes for a cytosolic GS1 isoform, GS1.5, and is reported to be mainly expressed in roots (Li et al., 1993). However, its precise function in the cell is still unknown (Martin et al., 2006). *ZmGln1.5* displayed a low level of expression in F44 compared to B73 (*P* < 0.005). This difference in gene expression was not supported by total GS activity in roots, however, which was found to be not significantly different between the two inbreds.

Relative root and shoot NR activity is highly variable within and across plant species (Andrews, 1986). In maize, previous studies have indicated that both root and shoot NR activities are dependent on the plant genotype, growing conditions (i.e., field vs. controlled environments) and the external nitrate concentrations provided to the roots (Reed and Hageman, 1980a,b; Andrews, 1986). In general, root NR activity is known to dominate when external nitrate concentrations are low (1 mM), while shoot NR becomes more important as external concentrations increase, as would occur with high rates of N fertilization (Andrews, 1986). For plants that show root NR activity, glutamine and asparagine are often abundant in the xylem sap while higher shoot NR activity is associated with greater pool sizes of glutamate, glutamine, aspartate, and alanine (Riens et al., 1991; Winter et al., 1992; Lohaus et al., 1998).

### Loss of the F44 Phenotype in the B73 × F44 Hybrid

In general, the F1 hybrid (B73 × F44) showed no heterosis and grew to a similar size and architecture than B73 (**Figure 8A**). B73 × F44 displayed analogous enzyme activities (**Figure 8E**), and had similar nitrate and ammonium uptake capacities to its B73 parent but not F44 (**Figures 8B,C**). Inbred crosses that display heterosis (e.g., a F1 hybrid of B73 × Mo17) often show gene expression levels statistically within the range of either of the two parents and frequently at intermediate mid-parent levels (Stupar and Springer, 2006). In this cross, we observed non-additive gene expression profiles in the F1 hybrid, a response previously observed in some maize hybrids (Song and Messing, 2003; Auger et al., 2005). Given the degree of variation between the inbreds at the level of gene expression, enzyme activities and nitrogen uptake patterns, a study of the recombinant inbred lines derived from the cross between B73 × F44 could yet lead to the identification of new quantitative trait loci indispensable to the understanding and improvement of NUE in crops. Regardless of the behavior of the F1, transgressive segregation in subsequent generations should allow mapping and cloning of the genes involved in NUE in maize.

## Conclusion

Maize, which produces more grain globally than any other crop, is used for human food, animal feed and more recently as an ethanol feedstock. Improvements to maize NUE will ultimately help deliver sustainable N fertilizer use in agriculture (Kant et al., 2012). We identified two contrasting maize inbreds, which differ in root architecture, and the transport and assimilation of nitrate and ammonium. The hybrid between the two lines exhibited the phenotype of only one parent, pointing to complete dominance of the B73 root genotype. Development of recombinant inbred lines from this cross will make it possible to identify, map, and isolate the enhancers or suppressors of root architecture as well as ion transport machinery. The study has highlighted that the root architecture may be an important variable in designing N efficient maize lines and in managing soil N acquisition based on soil type and availability of N across the soil profile. As maize germplasm improvement programs target improved NUE traits, this study provides unique insight into the link between RSA and N management and how both variables need to be explored together when defining NUE improvements in any selection program.

## Materials and Methods

### Plant Material and Growth Conditions

*Zea mays* inbreds, B73 and F44, were selected from a previously described set of diverse inbred lines (Liu et al., 2003; Garnett et al., 2015). Plants were grown in either an ebb and flow hydroponic system or in aerated 3.5 L pots in temperature controlled growth chambers. Seeds were imbibed in bubbling reverse osmotic water for 4 h before sown individually onto a supportive mesh within a polyvinyl chloride (PVC) seedling tube which contained moist autoclaved diatomite rocks (15 mm diameter). For phenotyping analysis, 4 days seedling tubes were transferred to pots containing 3.5 L of nutrient solution and grown in a controlled environment room with a day/night cycle of 14 h/10 h, temperatures of 28 and 23°C, respectively, and a light intensity of 300 μmol.m^-2^.s^-1^ at canopy level. For all other analysis, 4 days seedlings were transferred to a 700 L ebb-and-flow hydroponic system that allows for continual 15 min fill/drain cycles. The seedling tubes were placed within larger tubes (300 mm × 50 mm), which kept the roots of adjacent plants separate, but allowed for free access of the roots to nutrient solution. The hydroponic system was situated in a controlled environment room with a day/night cycle of 12 h/12 h, temperatures of 28 and 21°C, respectively, and a light intensity of 300 μmol.m^-2^.s^-1^ at canopy level. The plants were supplied with nutrient solution containing 2.5 mM NH_4_NO_3_, 0.5 mM MgSO_4_, 0.5 mM KH_2_PO_4_, 1.05 mM KCl, 1.25 mM K_2_SO_4_, 0.25 mM CaCl_2_, 1.75 mM CaSO_4_, 0.1 mM Fe-EDTA, 0.1 mM Fe-EDDHA, 25 μM H_3_BO_3_, 2 μM MnSO_4_, 2 μM ZnSO_4_, 0.5 μM CuSO_4_, and 0.5 μM Na_2_MoO_4_. The nutrient solution was changed weekly to maintain nutrient levels and solution pH around 5.9.

For root architecture analysis, the plants were grown on PVC pipes (100 mm wide × 1 m high) filled with one volume of diatomite rocks and two volumes of Nepean river sand under the same growing conditions as above. The plants were watered three times a week with 250 mL of the nutrient solution described previously.

### Root Measurements

After 4 weeks in the PVC pipes, the roots from five individual plants were separated from the shoots, then split between the four root types (primary, seminal, crown, and brace) and analyzed. The different roots were individually floated in water on a transparent plastic tray and imaged with an Epson perfection V700 photo scanner (Epson Australia Pty. Ltd., Australia). Images were analyzed using the WinRHIZO Pro 2012 software (Regent Instruments Inc., Canada) then dried and weighed.

### Gene Expression Analysis

After 21 days in the ebb-and-flow system, the roots were separated from the shoots, harvested and snap-frozen in liquid nitrogen. Total RNA was isolated using the TRIzol Reagent (Life Technologies, Carlsbad, CA, United States) according to manufacturer’s protocol. RNA concentrations were estimated using a NanoDrop ND-1000 Spectrophotometer (Thermo Scientific, Waltham, MA, United States). cDNA was generated using 1 μg of total RNA using SuperScript III Reverse Transcriptase kit (Life Technologies, Carlsbad, CA, United States). cDNA was mixed with TaqMan OpenArray^®^ Real-Time PCR Master Mix (Life Technologies^TM^, Carlsbad, CA, United States). All samples were run on a QuantStudio 12K Flex Real-Time PCR (RT-PCR) System (Life Technologies, Carlsbad, CA, United States) using 48-well plates TaqMan^®^ OpenArray^®^ RT PCR Inventoried Format 112 (Life Technologies^TM^, Carlsbad, CA, United States). Four genes, Ubiquitin-conjugating enzyme (*ZmUBQc*), SIN3 component, histone deacetylase complex (*ZmSIN3*), Cullin (*ZmCullin*) and Elongation factor 1-alpha (*ZmElF1*), were chosen as housekeeping genes to normalize gene expression data.

### Nitrate, Nitrite, and Ammonium Measurements

Nitrate, nitrite, and ammonium ions were extracted in 1 mL water from 3 to 4 mg of dried aliquots of plant material. Nitrate content was measured with a colorimetric method based on the detection of a chromophore obtained by reduction of nitrate to nitrite by Vanadium (III), adapted from Miranda et al. (2001). The nitrite contents were detected using the Griess assay (Wang et al., 2007). Ammonium was detected using the Ammonium Assay kit from Sigma-Aldrich (St. Louis, MO, United States, cat#AA0100) following the manufacturer’s protocol.

### Enzyme Activities

Nitrate reductase activity was measured according to Baki et al. (2000) using 300 mg of fresh material extracted in 1 mL extraction buffer.

Proteins were extracted from 200 mg of fresh material in 1 mL chilled extraction buffer [50 mM HEPES pH 7.5, 20% glycerol, 1 mM EDTA, 1 mM EGTA, 0.1% Triton X-100, 1 mM Benzamidine, 1 mM 6-Aminohexanoic acid and 1% Protease Inhibitor Cocktail (Sigma-Aldrich cat#P9599)]. After centrifugation, the clean supernatant was used to test the enzyme activities.

Glutamine synthetase transferase activity was measured by mixing 20 μL of extracted proteins with 80 μL of a reaction buffer (100 mM hydroxylamine, 125 mM MOPS, 0.5 mM ADP, 12.5 mM sodium arsenate, 37.5 mM glutamine and 1.25 mM MnCl_2_). After 30 min at room temperature, 100 μL of detection buffer was added (370 mM FeCl_3_, 576 mM HCl, and 157 mM TCA) and the optical density measured at 540 nm. L-glutamic acid γ-monohydroxamate (GHA) was used as a reference standard.

Nitrite reductase activity was measured by mixing 10 μL of extracted protein solution with 90 μL of reaction buffer (130 mM K_2_HPO_4_, 70 mM KH_2_PO_4_, 0.5 mM KNO_2_, and 2 mM methylviologen) and 10 μL of a sodium-based reaction solution (20 mg/mL of Na_2_CO_3_ and Na_2_S_2_O_4_). After 15 min at 30°C, the mixtures were vortexed to stop the reaction and the nitrite content determined using the Griess assay (Wang et al., 2007).

Asparagine synthase activity was measured by mixing 25 μL of extracted protein solution with 25 μL of reaction buffer (100 mM Tris-HCl, 10 mM glutamine, 20 mM aspartate, 20 mM MgSO_4_, and 10 mM ATP). After 30 min at room temperature, 20 μL of 0.2 M *N*-ethylmaleimide was added, mixed and then incubated for 10 min at 95°C and then placed on ice to cool. In the dark, 70 μL of the mixture was mixed with 100 μL of detection buffer [100 mM tricine, 1.8 mM NAD, 0.3% triton X-100, 0.15 U diaphorase and 0.6 mM MTT (Sigma M2128)] and the optical density of the solution read at 570 nm. The reaction was then started via the addition of 10 μL L-glutamic dehydrogenase (Sigma-Aldrich cat#G2626 – diluted ten-fold in 10 mM tricine). After 1 h at room temperature under light, the optical density was read again at 570 nm. Glutamic acid was used as the standard reference.

### Flux Measurements

After 21 days of growth in the ebb-and-flow system, nitrogen uptake capacities were measured as a unidirectional influx measurement in the middle of the day (11:00 am – 2:00 pm). Whole plants were transferred to a ^15^N-labeled nitrate or ammonium nutrient solution for 5 min. After the uptake period, the plants were transferred to a wash solution (nutrient solution without ^15^N) to desorb ^15^N label from the cell walls and then harvested. The ammonium ^15^N enriched solutions contained either 25 μM or 1.25 mM (^15^NH_4_)_2_SO_4_ (HATS or LATS measurements, respectively) whereas the nitrate ^15^N enriched solutions contained either 50 μM or 2.5 mM K^15^NO_3_ (HATS or LATS measurements, respectively). Roots and shoots were then separated, weighed and then dried at 60°C for a week, after which the roots were ground to a fine powder. Total nitrogen and ^15^N contents in the plant samples were determined with an isotope ratio mass spectrometer (Sercon, Crewe, Cheshire, United Kingdom). The nitrogen influx was calculated from the total N and ^15^N contents of the root.

### Experimental Design and Statistical Analysis

In general, independent experiments were conducted at least 2–3 times with data presented as a representative experiment. In all experiments, there were at least 3–5 individual plants grown, harvested and analyzed. Data is represented as mean ± SE (*n* = 3–5) of a single representative experiment. The statistical analysis of the experimental data was assessed using the Student’s *t*-test. Statistical significance was accepted when the probability of the result assuming the null hypothesis (*p*) is less than 0.05. Data described as significant is identified as ^∗^*P* < 0.05, ^∗∗^*P* < 0.01, ^∗∗∗^*P* < 0.005.

## Author Contributions

JD, KD, ST, JR, and BK contributed to the experimental design. JD and KF performed the experiments. JD and BK wrote the manuscript.

## Conflict of Interest Statement

The authors declare that the research was conducted in the absence of any commercial or financial relationships that could be construed as a potential conflict of interest. The reviewer LP and handling Editor declared their shared affiliation.

## Footnotes

1 http://ensembl.gramene.org/

## Supplementary Material

FIGURE S1 Expression of glutamine synthetase genes (*ZmGLN*) in roots of B73 (white) and F44 (black). Values are means (±SE) from three individual plants. Similar results were obtained in another independent experiment.

FIGURE S2 Expression of *ZmNRT3.1A* **(A)** and *ZmNRT2.1* **(B)** in B73 (white) and F44 (black) in the primary (PR), seminal (SR) and crown (CR) root tissues. Values are means (±SE) from 4 to 5 individual plants. Different letters indicate significant differences among means at *P* < 0.05 (ANOVA).

FIGURE S3 Comparison of BK3 (gray) seminal **(A)** and primary **(B)** root phenotypes with the parents B73 (white) and F44 (black). The data were normalized to B73. Values are means (±SE) from five individual plants. Asterisks indicate significant differences with B73 at ^∗^*P* < 0.05, ^∗∗∗^*P* < 0.005 (Student’s *t*-test).

FIGURE S4 Expression of nitrogen transporter and metabolism pathway genes in B73 (white), BK3 (gray) and F44 (black) roots. The data were normalized to B73. Values are means (±SE) from three individual plants. Similar results were obtained in another independent experiment. Asterisks indicate significant differences with B73 at ^∗^*P* < 0.05, ^∗∗∗^*P* < 0.005 (Student’s *t*-test). (NPF/NRT, nitrate transporter; AMT, ammonium transporter; GLN, glutamine synthetase; NIR, nitrite reductase; GOGAT, glutamate synthase; GDH, glutamate dehydrogenase; n.d., non-detectable).

TABLE S1 List of genes studied in this paper.
