# Supplementary figures and images for: Root Ideotype Influences Nitrogen Transport and Assimilation in Maize
Source: Front Plant Sci. 2018 Apr 24;9:531. doi: 10.3389/fpls.2018.00531 (PMC5928562; doi:10.3389/fpls.2018.00531)

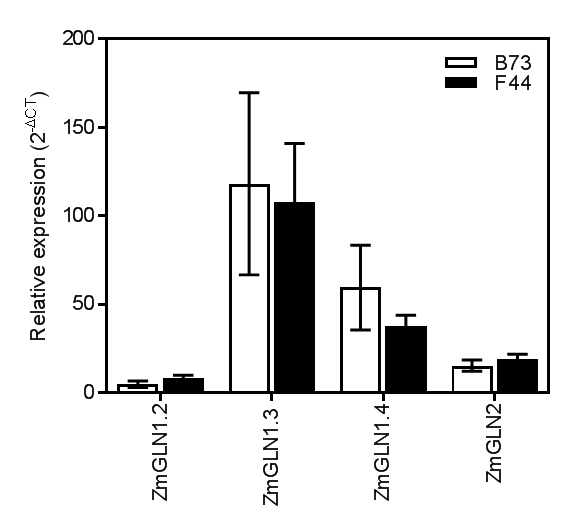

Supplement: FIGURE S1 — Expression of glutamine synthetase genes (ZmGLN) in roots of B73 (white) and F44 (black). Values are means (±SE) from three individual plants. Similar results were obtained in another independent experiment. [file Image_1.TIF]

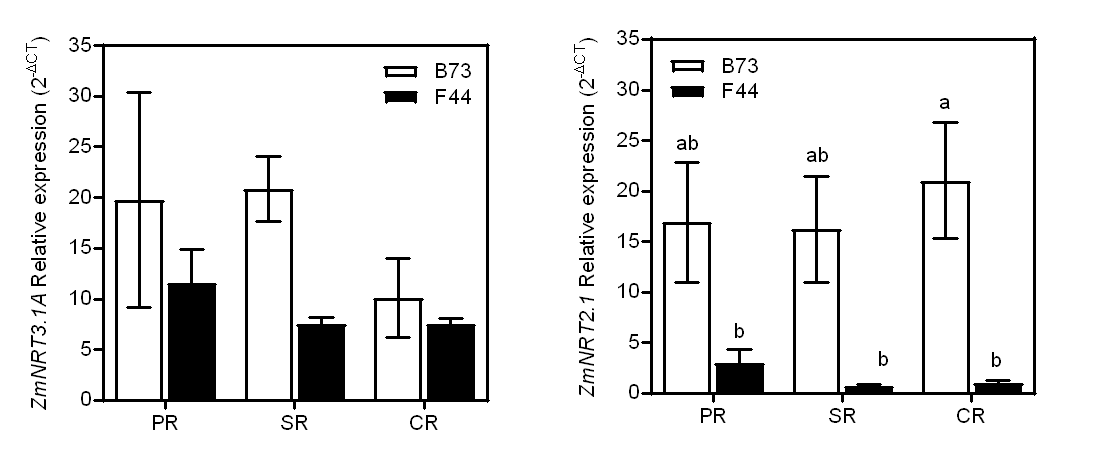

Supplement: FIGURE S2 — Expression of ZmNRT3.1A (A) and ZmNRT2.1 (B) in B73 (white) and F44 (black) in the primary (PR), seminal (SR) and crown (CR) root tissues. Values are means (±SE) from 4 to 5 individual plants. Different letters indicate significant differences among means at P < 0.05 (ANOVA). [file Image_2.TIF]

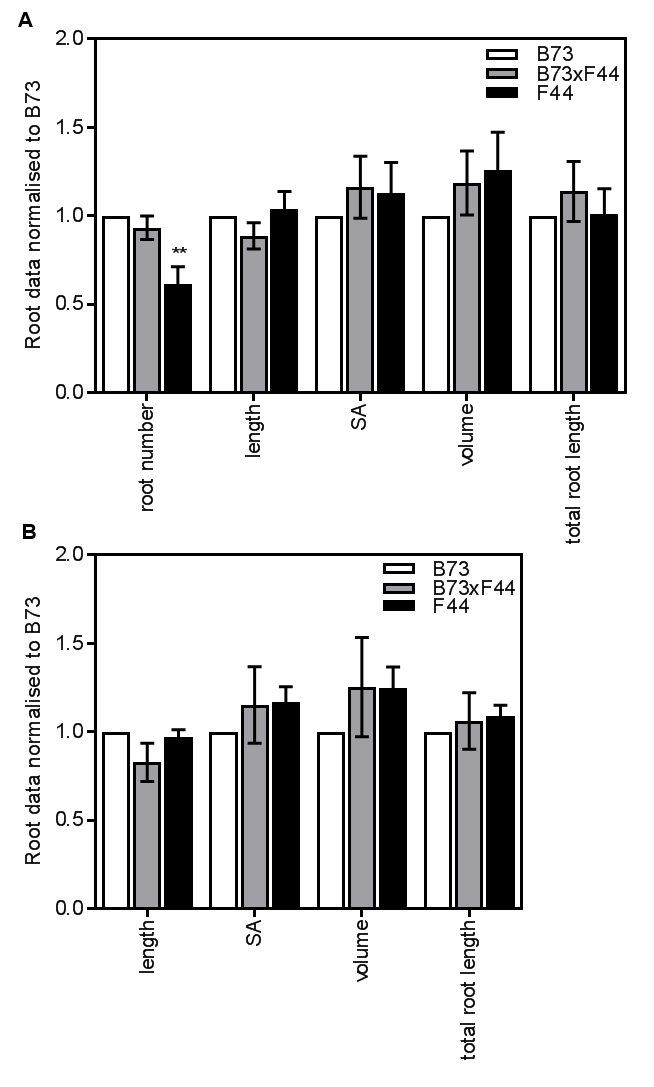

Supplement: FIGURE S3 — Comparison of BK3 (gray) seminal (A) and primary (B) root phenotypes with the parents B73 (white) and F44 (black). The data were normalized to B73. Values are means (±SE) from five individual plants. Asterisks indicate significant differences with B73 at ∗P < 0.05, ∗∗∗P < 0.005 (Student’s t-test). [file Image_3.TIF]

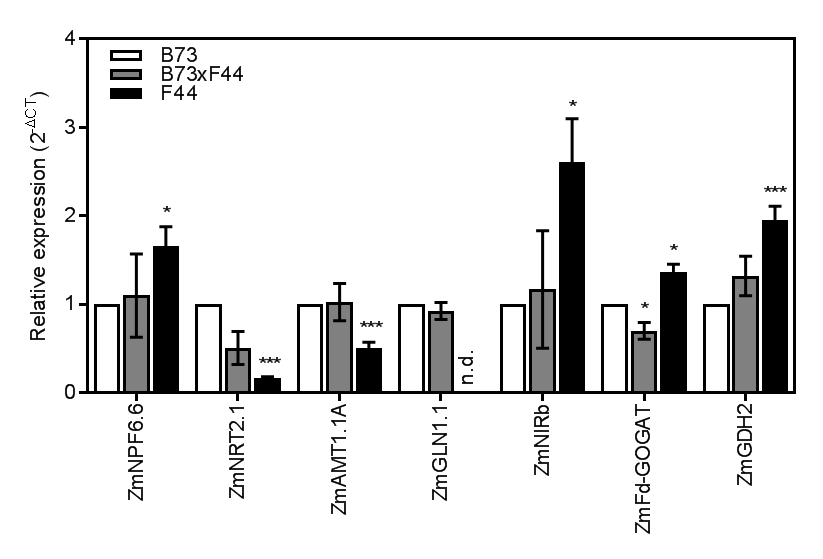

Supplement: FIGURE S4 — Expression of nitrogen transporter and metabolism pathway genes in B73 (white), BK3 (gray) and F44 (black) roots. The data were normalized to B73. Values are means (±SE) from three individual plants. Similar results were obtained in another independent experiment. Asterisks indicate significant differences with B73 at ∗P < 0.05, ∗∗∗P < 0.005 (Student’s t-test). (NPF/NRT, nitrate transporter; AMT, ammonium transporter; GLN, glutamine synthetase; NIR, nitrite reductase; GOGAT, glutamate synthase; GDH, glutamate dehydrogenase; n.d., non-detectable). [file Image_4.TIF]
