# Supplementary material for: Root Ideotype Influences Nitrogen Transport and Assimilation in Maize
Source: Front Plant Sci. 2018 Apr 24;9:531. doi: 10.3389/fpls.2018.00531 (PMC5928562; doi:10.3389/fpls.2018.00531)
Supplement: TABLE S1 — List of genes studied in this paper. [file Table_1.docx]

*Supplementary Table 1*

List of genes studied in this paper

| **Name** | **Genome ID** |
| --- | --- |
| *ZmNPF6.4* | GRMZM2G086496 |
| *ZmNPF6.6* | GRMZM2G161459 |
| *ZmNPF6.7* | GRMZM2G112154 |
| *ZmNPF6.5* | GRMZM2G161483 |
| *ZmNRT2.1* | GRMZM2G010280 |
| *ZmNRT3.1A* | GRMZM2G179294 |
| *ZmAMT1.1A* | GRMZM2G175140 |
| *ZmAMT1.3* | GRMZM2G028736 |
| *ZmAMF1.1* | GRMZM2G062024 |
| *ZmAMF1.2* | GRMZM2G164743 |
| *ZmGLN1.1* | GRMZM2G050514 |
| *ZmGLN1.2* | GRMZM2G024104 |
| *ZmGLN1.3* | GRMZM5G872068 |
| *ZmGLN1.4* | GRMZM2G036464 |
| *ZmGLN1.5* | GRMZM2G046601 |
| *ZmGLN2* | GRMZM2G098290 |
| *ZmNIAa* | GRMZM2G428027 |
| *ZmNIAb* | GRMZM5G878558 |
| *ZmNIAc* | GRMZM2G568636 |
| *ZmNIAd* | GRMZM2G076723 |
| *ZmNIRa* | GRMZM2G079381 |
| *ZmNIRb* | GRMZM2G102959 |
| *ZmFd-GOGAT* | GRMZM2G036609 |
| *ZmGDH1* | GRMZM2G178415 |
| *ZmGDH2* | GRMZM2G427097 |
| *ZmAsnS1* | GRMZM2G074589 |
| *ZmAsnS2* | GRMZM2G093175 |
| *ZmAsnS3* | GRMZM2G053669 |
| *ZmAsnS4* | GRMZM2G078472 |
| *ZmUBQc* | GRMZM2G027378 |
| *ZmSIN3* | GRMZM2G334457 |
| *ZmCullin* | GRMZM2G166694 |
| *ZmElF1* | GRMZM2G154218 |
